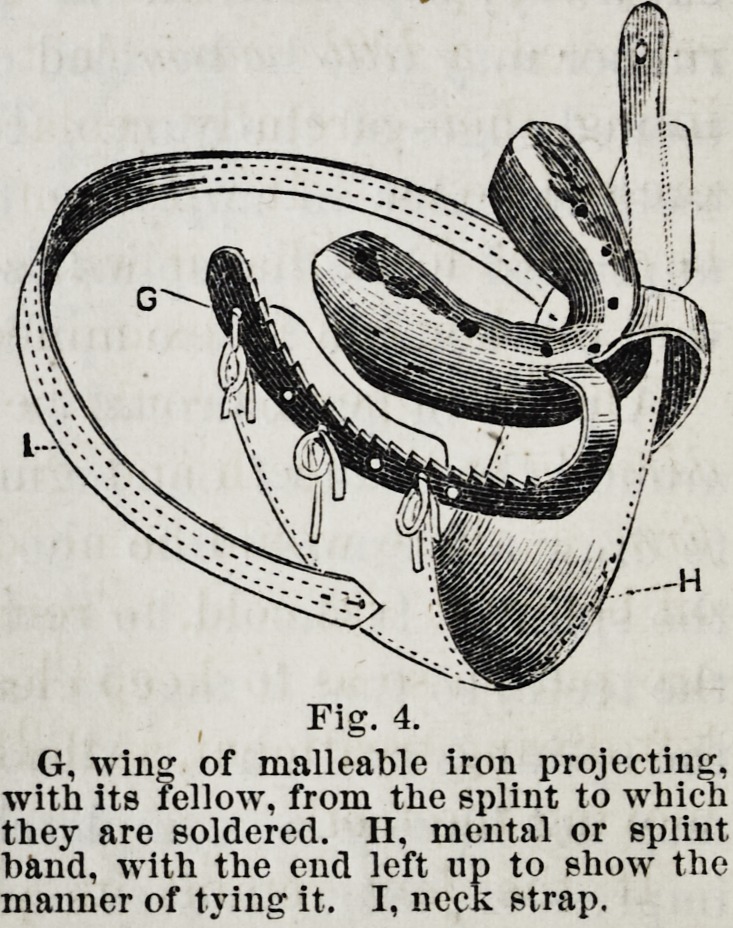# Treatment of Fracture of the Lower Jaw by Interdental Splints

**Published:** 1868-07

**Authors:** Thomas Brian Gunning


THE
AMERICAN JOURNAL
OF
DENTAL SCIENCE.

Vol. 11. THIRD SERIES--JULY, 1868. No. 3.

### ARTICLE I.

### *Treatment of Fracture of the Lower Jaw by Interdental Splints.*

(CONTINUED.)

By THOMAS BRIAN GUNNING.

Dental works give full directions for the vulcanization of rubber, and also as to many things necessary to a successful application of these splints.

Before applying the splints, all the projections caused by air holes, or other imperfections in the plaster-cast, must be cut away, especially in the parts covering the teeth. The rubber may also be beveled off where it fits close on the festooned edges of the gum. This will give more room for the teeth to enter in applying the splint, and leave the gum unpressed while the splint is worn. The latter should be well oiled inside before application.

A piece of pack-thread or silk, about a foot long, placed around the neck of one or more teeth, is frequently useful to draw a fragment into the position suitable for entering the splint. It should be tied at the ends, but not around the teeth, so that it may be easily cut and drawn away before the splint is on tight. Although the fragments of the bone may not have gone completely into place before taking the impression, little anxiety need be felt as to their going up into the splint if the latter has been properly adjusted, as the muscular displacement frequently yields to the more normal condition produced by the splint, even when it is only partially in place.

If the jaw should not go well up in the splint, it may be worn loose for a day or two, to allow the muscles to relax. This, however, is rarely necessary.

A tube, just large enough to slide into the female screws, should be inserted, to protect them while the teeth are being drilled to receive the ends of the screws. The tube must be made of thin plate, and should be set at a right angle in the end of a thick piece of plate, that the latter may serve as a handle to keep the tube from turning with the drill.

Rubber splints are neat and comfortable. They can be kept free from food and all unpleasant odors, if frequently cleaned externally with a tooth brush, and in the inside of the mouth by means of a small sponge on the end of a crooked probe, when the jaws are fastened together. They should also be frequently syringed with warm water, etc.

Fig. 4. This splint is made of tin. Six or eight sizes might be cast (and kept ready for use,) from which one could be selected suitable for the jaw. The wings are of malleable iron, tinned to prevent rusting and for more readily soldering. Three sizes would be sufficient to select from.

The splint should have a handle in front, that it may be used as a cup to take the impression of the jaw—the holes being useful to allow a small probe to be pressed through the wax down to the teeth, thus allowing air to enter to facilitate the removal of the impression, and when in use as a splint giving entrance to warm water, thrown from a syringe, to keep the parts clean.

Fig. 4.

G, wing of malleable iron projecting, with its fellow, from the splint to which they are soldered. H, mental or splint band, with the end left up to show the manner of tying it. I, neck strap.

The splint should be made to fit well by bending, cutting off the edges and rounding them up smooth. When a tooth projects so as to keep the splint from fitting, a hole may be cut to let the tooth through, if the metal cannot be hammered out. This should all be done before taking the impression, as a well fitted cup assists greatly in this important matter.

(The adaptability of this splint is shown in the fact that the one from which the cut was taken had been used successfully on two different jaws, so unlike that the first was a quarter of an inch wider, where the ends of the splints rested, than the second. When fitting it to the second jaw, it was necessary to cut off a part of the right wing, to keep it clear of the corner of the mouth. This accounts for the difference in the width of the arches as seen in the cut. The indentations on the top of the splint were made by the boys in eating.)

After the *cast* is obtained, the handle in front should be cut off, and the wings, *if needed*, soldered on, care being taken that their edges are clear of the corners of the mouth, when *open*. Warm gutta-percha should then be placed in the splint, pressed down on the cast, and, after cooling it in water, dig out the softened plaster.

If the splint is found to rock on the teeth, it should be removed, a *little warm* (not hot) *water* be poured into the lining, then carefully replaced upon the teeth, and slightly pressed down. It will then fit perfectly. This lining will be of such form that it will come off the teeth readily, therefore the jaw can be examined when desirable.

The gutta-percha could be placed in the splint and applied *directly* to the teeth and gum, if the jaw is set *sufficiently firm*, as there would be no difficulty in drawing the lining off before it was cold, to remove the ligatures. But if they are put on so as to keep clear of the gum, they might be left during treatment, as the lining would prevent them from moving the teeth.

If the jaw retains its place when the gutta-percha is pressed down, the splint might be *left* on. In this way the gutta-percha, by embracing the teeth, and fitting in between them, would hold the fragments of the jaw firmly in place.

It is, however, much more difficult to apply gutta-percha than wax, as it requires more heat and pressure.

When the jaw can be just held in place and will bear but little pressure, hardly that of warm wax, plaster of Paris might be used as a lining. In many cases it would hold the fragments in the splint for a long time.

This splint can be used without wings, in any way that Fig. 1 will answer.

The mental or splint band must be used when there are no teeth suitable to fasten to. This is frequently the case in children. This band may be removed for washing when necessary, care being taken that the patient keeps the jaws closed during the removal, in the earlier stages of treatment.

The splint has so far been spoken of in its adaptation to fractures in which the jaw is allowed to *move*. It can also be used instead of Figs. 2 and 3, by soldering suitable portions of another splint on the upper part, to hold the lining for the upper teeth. When the teeth are not fit for screws, the cap of Fig. 3 could be used, with long tapes to reach down to the wings beside the lower jaw, if a ready-made lower wing could not be fitted so as to act in place of an upper one.

No care will keep this splint as pleasant as one made of rubber. Gutta-percha absorbs, and becomes very offensive, but the small quantity used for lining the splint is protected and covered so that, with great cleanliness, it may be worn with little annoyance.

This splint has the advantage of being easier of application, and can be applied, if ready made, in much shorter time than a rubber splint.

In fractures treated with either kind of splint, the trouble and anxiety are over when the splint is on, as there is then no chance for the jaw to get misplaced.

In ordinary cases the splints may be removed during the first three days, if any edge is pressing so much into the gum as to be painful. With proper care in the fitting this will be unnecessary.

These splints hold the fragments so well together that I have seen badly lacerated gums heal up so perfectly in from two to three days, that the fractures were then only simple.

No bad effects are produced by splints covering the teeth and gum. On the contrary, teeth that are so much loosened by the injury as to be beyond recovery in the usual treatment, are securely held by the splint and become firm again. The gum looks red and soft while the splint is worn but a short period suffices for its complete restoration, even when it has been covered up for months. I generally leave the splint on long enough to feel assured that temporary removal will not endanger the union, which is very delicate for some time. How soon this will be, after the first application of the splint, and how long before the splint can be dispensed with, depend upon the gravity of the injury and the state and age of the patient.

With the fragments held in place, little apprehension need be felt of those painful abscesses, exfoliations and other complications so often present in the usual treatment. The advantages of splints over bandages are so great that nothing but experience will give a full appreciation of them to any one. I am able to speak positively upon this point, as nearly all the cases treated by me had been found unmanageable by the old methods, before coming under my care, and some of them were gravely complicated.

The following are examples:

Case 1.—A seaman, senseless from explosion of powder on board a Spanish frigate, was sent to the U. S. Naval Hospital. A comminuted fracture of the lower jaw was found between the canines, a piece of the bone loose in the mouth, the teeth of both jaws much shattered, with face severely burnt and lacerated. The case had been carefully treated for over four months without producing any union, when, by the advice of Surgeon Bache, Director of the Naval Laboratory, I was requested to treat it. The jaw was contracting from loss of bone, and pieces were coming out through the chin. I applied a hard, vulcanized rubber splint, which inclosed the remaining teeth and gum of the lower jaw, its upper surface fitting well over the teeth above, except in front, where it was trimmed down to allow food to pass between the remnants of the superior incisors. The splint was fastened to the lower jaw by screws passing into a broken tooth on each side. The jaw was held up by starched muslin, moulded to a cast of the parts, in repeated folds, until a line in thickness.† This reached to the zygomas, and was kept up by a band passing over the head. The splint was applied Feb. 12, 1861. Fragments of the bone came away for some time after, but the splint was not removed during the treatment. The jaw united well by the middle of May, and the man was sent home to Cuba.

Splints of similar construction, but without screws, and with a different bandage, were subsequently used with great success in over forty cases in one of the hospitals of the Confederate army in 1864.‡

Case 2.—I received a compound fracture in my own jaw between the right canine and lateral incisor teeth on November 4th, 1862, through my horse falling under me. The bone was much displaced and two incisor teeth loosened. I set the bone, and it was held by strong, well stretched silk, inclosing three incisors, the right canine and first bicuspid. This stopped the bleeding forthwith and held the bone firmly. A vulcanite splint was applied thirteen hours after injury. It inclosed all the lower teeth, and was fastened by gold screws to the first molars. It held the fragments so well that I was able to attend to patients in the afternoon,

† A bandage of thick gutta-percha was tried first, but it yielded to the shape of the jaw so much that it increased the tendency to contraction. The pliancy of gutta-percha is a radical objection to its use in or out of the mouth, except when it can be supported.

‡ See Richmond Medical Journal, Feb. 1866.

and continued to do so subsequently. The gum united by first intention, and the pain and swelling, which were very great in the external parts, diminished rapidly.

November 28th the splint was removed, and good but flexible union found. It was again fastened on, but after seven days was worn without the screws, and removed daily. The jaw grew strong, the teeth firm, and the splint was left off January 1st, 1863, but worn at night until February 1st. Jaw was used in eating, talking, etc., throughout the treatment. The incisor teeth have regained their communication with the inferior dental nerve. This was severed by the displacement of the fragments, which was so great as to admit the little finger between the teeth. Judging from the sensation of slight tightness between the front teeth in certain movements of the muscles, the bone was twelve months in growing as stiff as before the accident. The case was presented to the New York Academy of Medicine, January 7, 1863, by Dr. A. L. Sands. Prof. Alexander Stevens said the splint was a great improvement, and that the treatment would last forever.† The splint was brought before the Medical Society of the State of New York in February.‡

Case 3.—G. B., forty-five years old. Jaw fractured through socket of right second bicuspid, June 5, 1863, by a blow. Displacement of back fragment inward and forward. Patient could not lie down, but slept in a chair, *holding* the jaw, as the surgeons could not keep the fragments in place. The fracture commenced inside the first bicuspid tooth, and passed backward and outward through the socket of the second, and downward also, at the expense of the back fragment. As the loosened bicuspid had been extracted instead of being kept in place, there was nothing to prevent the back fragment from sliding inward and over the front one. It was set and held in place by a jackscrew, of which one end rested on the left side, between the first and second molar teeth. The other end went into the short fragment, about the centre of the fracture, and as low down

† See Bulletin of the Academy.

as the muscles under the tongue allowed. This held the parts firm while the impression and bite were taken, the mouth cup being notched out to go down over the ends of the jackscrew. On June 17th I applied a splint like Fig. 1, without screws, but held down by a strip of silk passing under the chin, and, supported by wings which projected from the splint, came out over the lower lip, and continued along the sides of the jaw like the wings of Fig. 4. Splint held the bone in place, although there were but two loose teeth in the back fragment—first molar having been out for years and the second bicuspid lost through the fracture. Patient could now lie down comfortably. The band was worn snug until June 24, when it slackened because of painful swelling under the chin. No displacement following, the band was worn loose afterward. July 20th, splint removed to examine the jaw and flexible union found; 29th, callus firmer. August 8th, improving; 18th, wings cut off, but splint worn until September 3d. Jaw allowed its natural motions throughout treatment.

This splint was presented to the New York Academy of Medicine, in October, 1863.† I received the thanks of the Academy, accompanied by a request to report further when I should have completed the splint which I considered best adapted for general use. In answer to this request, the splint represented by Fig. 4 was fully described in the paper mentioned at the head of this article.

Case 4.—J. Q., twenty-five years of age, had his jaw broken by being thrown from a cart, December 29th, 1863. On the same day he called in a physician, who tied the teeth together and sewed up a deep gash over the left masseter muscle. The ligature did not permanently control the fracture; the teeth became very loose and the front of the jaw was drawn back inside of the left fragment. Patient went into the Bellevue Hospital January 9th, 1864. The left lateral incisor, loosened by the accident, having been extracted, attempts were made to hold the jaw in place by passing wire around the teeth, but without success. January 14th patient was brought to my office. I find the jaw fractured through the socket of the left lateral incisor, slanting toward the symphysis as it descends, thence back at the expense of the inside of the left fragment. The gum is red and painful; great tenderness under the jaw and upon the ramus, which was also *supposed to be fractured.* I find it is not. The gash across the left masseter muscle is about two inches long, and through it the bone can be distinctly felt with the finger; much swelling, which is extending; pus discharging freely into the mouth and externally from the wound near the angle. Tied the fragments, taking in the remaining incisors, both canines and left bicuspids in the ligature, as the central incisors were quite loose, the one next the injury and also the left canine so much so that the fingers would have taken them out easily. A piece of wood was placed endwise across the socket of the extracted lateral incisor, bearing against the central incisor and the canine, to prevent displacemeut while taking the impression. This held the fragments in place, but it was impossible to get the jaw into its natural position relatively to the upper. The left masseter muscle, weakened by the cut, having been inactive for so long a period, the parts had settled over the left, and I was obliged to take the bite in that position.

January 15th. Applied a vulcanite splint, like Fig. 1 without screws or any other fastening. It held the fragments in place, and the patient experienced great relief. February 13th, took off the splint temporarily, no displacement followed, but union was very soft. After this, removed the splint and examined the parts weekly. March 19th, the wound is healed. Removed the necrosed socket of the extracted incisor. Union firmer, teeth improved. April 9th, union strong, but it is advisable to wear the splint longer on account of the canine tooth, which is growing firm. The jaw now articulates with the upper, and the upper and lower teeth fit against each other well. May 1st, splint dispensed with.

I have used this kind of splint on many patients, and always successfully. Amongst them were cases which had been treated, without avail, in civil and military hospitals of this and other places. I have never seen it fail to hold the bone in place, although used without any fastening in the mouth, or support externally from bandage. In one case the jaw was broken by a minnie ball into seven or eight pieces, and part of them, with one tooth lost.† In another much of the mental process was shot away, together with three front teeth.

Case. 5.—Mary Ann D., twenty-nine years old, was found in a state of insensibility, Feb. 12, 1864, and sent to the Bellevue Hospital the next morning. She remained unconcious until the 16th.

February 17th. Dr. R. B. Brownell spoke to me of her broken jaw, but said nothing could be done to it at present, as her head and face were so terribly swollen.

February 21st. Saw the patient at the hospital, and found her lower jaw broken on the right side, commencing half an inch back of the canine tooth, and passing downward at the expense of the back fragment. There have been no teeth back of the canine for some time, and, the gum being torn, the back fragment rides over the front, with its point sticking out sharp and bare, for three-eights ($\frac{3}{8}$)

---

† I extracted this tooth, the left central incisor, it being forced out in front as the lateral had closed up so as to touch the right central incisor through the contraction of the parts, the fracture being two months and ten days old when I took charge of the case. One fracture went down through the socket of the ejected tooth, and another between the second left bicuspid and fiirst molar. The alveolar inside the four teeth between these fractures was all necrosed, and that outside completely loosened from the bone below, the separation being horizontal and on a line with the end of the roots. This alveolar, with the four teeth attached to it, would have turned down externally at a right angle had the gum been cut vertically at the ends. I took away the necrosed portion, made the outer part fit at the symphysis, and set all in place. The splint was applied July 22d, 1864. When it was taken off December 11th, the jaw was united in every part, and the teeth were all fast with the gum firm around them, but on the inside not quite as high as on the corresponding teeth of the other side of the mouth. To avoid being sent to the army again, the man wore the splint three months longer, without my knowledge, but the teeth and gums were not injured any by it.

of an inch, in the direction of the symphysis. Although there is much swelling around the fracture, in and out of the mouth, also over the left zygoma and down the ramus, there is *great mobility* of the front of the jaw.

February 22d. Patient was brought to my office. Swelling on the face lessened somewhat, but still undiminished in the gum around the fracture. On the left side there are no teeth back of the bicuspid, and the gum is sound and healthy, but indented by the upper wisdom tooth which has been pressing into it since the accident, previous to which it had not done so, except when the gum was swollen eighteen months before. This condition of parts induced me to examine the left ramus carefully, and I found great play of its upper back portion, especially inward, but the only displacement when at rest, is upward and forward, and this to no great extent, as it is checked by the upper wisdom tooth. Finally concluded that a fracture exists in the neck of the condyle, passing downward and backward, thus allowing the muscles to draw the bone upward and forward.

The lower jaw contains only the four front teeth, the two canine and first left bicuspid. The gum back of these is free from roots, except that of the right wisdom tooth, which still remains, but decayed close down. The upper jaw has been without the eight teeth forward of the second bicuspids for some time; of the other eight, seven still remain, the right second molar only having been extracted.

To set the jaw, the right fragment was put in the best position that could be obtained with the fingers, assisted by a stout piece of silk passing around the left canine. A jack-screw, with a collar fitting against the root of the wisdom tooth in right fragment, and the other end bearing on the gum between the left lateral incisor and canine teeth, was then screwed out until the extension was sufficient to allow the fractured bone to come into proper position. The end of the long or forward fragment was then held up, and an impression in soft wax taken of all the teeth and gum, as far back as the ramus on each side. Care was taken to put the bone in place at the neck of condyle while the bite was obtained.

February 28th. Applied the splint.† The surgeon who brought the patient to my office wished to try and hold the chin up with a leather bandage of Hamilton's pattern. It held the chin up very well for a short time, when tightly buckled, but in an hour the jaw fell away somewhat.

February 29th. Swellings on the head, temples, etc., with pain caused by the bandage.

Compresses were placed over the head and temples, and great pains taken to prevent the bandages from hurting. It was worn so loose that the teeth went up and down in the splint to such an extent that it was feared the jaw would get out entirely.

March 2d. Patient brought a request from the surgeon in charge of her case at the hospital that I would screw the splint fast to the teeth, that the bandage may be dispensed with, for the swellings on the head, temples, etc., are much increased. The lower lip is also very painful on the right side in front of the canine. Gum has grown over the point of the bone; it is therefore only a simple fracture now. Screwed the splint fast.

March 10th. Swellings caused by bandage nearly gone. Patient complains of pain in swallowing. Removed splint, and shortened the left end, which had cut into the palato-glossus muscle. Bone is united so well as to keep its form, and the fracture at the neck of the condyle is doing well. No complaint as to the teeth.

March 14th. Patient in good spirits and quite comfortable. Wants to leave the hospital and go to work. No complaint as to teeth.

March 26th. At my request patient was discharged from the hospital, but still wearing her splint.

April 8th. Splint removed and good union found. Splint was worn just forty days, but the patient has a fine constitution and the bone united rapidly.

---

†See Fig. 2 taken from the original.

June 7th 1865. Patient sent me word that the jaw was all right.

In this case the fractured jaw was held by the splint in proper relative position to the upper jaw, but in the next case the jaw was held *out* of its proper position.

Case 6.—Patient thirty-six years old, the son of a physician in Brooklyn; jaw fractured through the symphysis, and the right condyle dislocated outward and backward, February 10th, 1866, in falling down stairs, and striking the chin on a small desk. The dislocation was reduced, but the displacement of the jaw being found uncontrollable, I was called in consultation.

February 14th.—Patient has been confined to his bed since the accident, motion being insufferably painful. The right side of the jaw is so much out of place that the lower back teeth strike nearly outside the upper. At the point of fracture, the left fragment is inside the right with a lateral displacement of five or six lines, and nearly that much vertical displacemeut. Much swelling and pain under and inside the front of the jaw, with terrible suffering in the right glenoid fossa and ligaments when the condyle is moved. There is a firm smooth swelling upon the outer part of the neck of the condyle, but nothing that indicates fracture of the bone, although the back teeth touch too soon, and it is impossible to get the lower bicuspids up to those above them. This is probably caused by some displacement or injury of the interarticular cartilage, which allows the condyle to go up too far into the glenoid fossa. The left side of the jaw will move in any direction, being uninjured, and the muscles in a good condition (except at the symphysis.) This accounts for the fragments being carried over to the right side, where the ligament and muscles are so *crippled* as to be *unable* to *balance*, or antagonize those in good condition on the left. Packthread was passed arouud the left bicuspid, with a peice of wood through the other end, to assist the fingers while the bone was drawn over to the left side. At the same time this fragment was pressed down with the fingers, aided by levers and wedges of wood. The muscular resistance to motion was so great that all efforts to bring the fragments into position were ineffectual for a long time, although the left half was drawn steadily over to its own side. But after two hours' effort the parts yielded sufficiently, and a piece of wood was fitted across the roof of the mouth, between the upper teeth, and extending under their crowns. Its lower surface was cut out to receive the teeth of both sides of the lower jaw, and the fractured ends, at the symphysis, were secured by thread passed around the teeth. The patient felt much relieved by this, although exhausted by the pain experienced in accomplishing it, as it was not thought advisable to give anæsthetics. Probably the parts would have come into place readily under their influence, but whether they could have been held there so well afterward is more doubtful.

February 15th. Patient walking round, feeling much better. The halves of the jaw are in comfortable position. The parts near the fracture have improved greatly since relieved from the pressure of the displaced ends of the bone, and the jaw opens wider. Took wax impression of upper and lower teeth, etc. The lower jaw being only imperfectly set, the plaster cast was sawed apart between the central incisors and adjusted by the upper cast. The packthread was still allowed to remain on the lower bicuspid, that the patient might draw the jaw into place, should it settle to the right again.

(*To be continued.*)

---

## Figures and Tables

**Fig. 4. f1:**